# Comparative study of indocyanine green (ICG)-R15 and Albumin-Indocyanine Green Evaluation (ALICE) grading system in the prediction of posthepatectomy liver failure and postoperative mortality in patients with hepatic alveolar echinococcosis

**DOI:** 10.1186/s12876-022-02367-x

**Published:** 2022-06-14

**Authors:** Yuxin Liang, Zilong Zhang, Zonglin Dai, Rui Cao, Deyuan Zhong, Chunyou Lai, Yutong Yao, Tianhang Feng, Xiaolun Huang

**Affiliations:** 1 Present Address: Department of Hepatobiliary-Pancreatic Surgery, Cell Transplantation Center, Sichuan Provincial People’s Hospital, University of Electronic Science and Technology of China, No. 32, West Second Section, First Ring Road, Qingyang District, Chengdu, 610072 Sichuan China; 2 Chinese Academy of Sciences Sichuan Translational Medicine Research Hospital, Chengdu, 610072 China; 3 The Second Department of Hepatobiliary Surgery, Sichuan Provincial People’s Hospital, University of Electronic Science and Technology of China, Chengdu, China; 4 Clinical Immunology Translational Medicine Key Laboratory of Sichuan Province and Organ Transplant Research Institute, Sichuan Provincial People’s Hospital, University of Electronic Science and Technology of China, Chengdu, 611731 China

**Keywords:** Hepatic alveolar echinococcosis (HAE), Indocyanine green (ICG)-R15, Albumin-Indocyanine Green Evaluation (ALICE) grading system, Posthepatectomy liver failure (PHLF), Postoperative mortality

## Abstract

**Background:**

A precise evaluation of liver reserve function in patients with hepatic alveolar echinococcosis (HAE) prior to hepatectomy could substantially increase the success rate of the operation and reduce the incidence of postoperative complications. The present study aimed to investigate the significance of the indocyanine green retention test at 15 min (ICG-R15) and the Albumin-Indocyanine Green Evaluation (ALICE) grading system in predicting severe posthepatectomy liver failure (PHLF) and postoperative mortality in HAE patients undergoing liver resection.

**Methods:**

A total of 105 HAE patients undergoing hepatectomy were enrolled in this study. The value of each variable in predicting severe PHLF was evaluated by univariate and multivariate logistic regression analyses. The area under the receiver operating characteristic (ROC) curves (AUC) were calculated to evaluate the predictive ability of the Child–Pugh grade, ICG-R15, and ALICE grading system. Also, patients were classified using the optimal cutoff value for ICG-R15 and different ALICE grades, and the incidence of severe PHLF and postoperative mortality were compared with the predicted values.

**Results:**

Out of the 105 HAE patients enrolled in this study, 34 patients (32.4%) developed severe PHLF. The ALICE grade and operative time were identified as independent predictors of severe PHLF. According to ROC analysis, the AUCs of the Child–Pugh grade, ICG-R15, and ALICE grade for predicting severe PHLF were 0.733 (95% confidence interval (CI), 0.637–0.814), 0.823 (95% CI, 0.737–0.891), 0.834 (95% CI, 0.749–0.900). The incidence of severe PHLF and postoperative 90-day mortality in patients with ICG-R15 > 7.2% were significantly higher than those with ICG-R15 ≤ 7.2% (*P* < 0.001; *P* = 0.008). Likewise, the incidence of severe PHLF and postoperative 90-day mortality in patients with ALICE grade 2 were higher than those with ALICE grade 1 within the Child–Pugh grade A (*P* < 0.001; *P* = 0.083).

**Conclusion:**

ICG-R15 and ALICE grading system are powerful predictors of severe PHLF and postoperative mortality among HAE patients undergoing hepatectomy. Furthermore, a combination of the preoperative Child–Pugh grade and ALICE grading system may provide an even more precise and objective guidance and facilitate surgical decision-making for HAE patients.

## Introduction

Hepatic alveolar echinococcosis (HAE) is a potentially lethal infectious disease or zoonosis caused by larvae of the tapeworm *Echinococcus multilocularis*. Although alveolar echinococcosis can parasitize in various visceral organs of the human body, the liver is the most common organ affected [1–4]. Among the 18,235 estimated new cases of human HAE recorded each year globally, 91% occur in China alone, mainly on the Tibetan plateau (in Qinghai, Sichuan, and Tibet) [5]. In cases of HAE, therapeutic decisions are based on the possibility of complete resection of the liver lesions, whereas curative hepatectomy is considered to be a safe treatment to eradicate the parasites and provide a permanent cure [6–8]. Moreover, for advanced cases affecting the major bile ducts and vessels (portal veins, hepatic veins, and vena cava), aggressive hepatic surgery is still recommended, though there is a higher risk of morbidity and mortality resulting from uncontrolled bleeding or liver failure [9]. Therefore, the surgical treatment of HAE patients requires a more sensitive and precise preoperative assessment than Child–Pugh grade, MELD (model for end-stage liver disease) score, and analysis of liver reserve function based on ICG-R15 alone to improve the probability of successful surgery, reduce postoperative complications.

The two most common methods to assess liver functional reserve are the Child–Pugh score and MELD score [10–12]. However, these static assessments are sometimes not sensitive enough to predict the postoperative liver function accurately. Therefore, another test called the indocyanine green retention test at 15 min (ICG-R15), a continuous and quantitative test for evaluating the liver blood flow and functional hepatocytes, is increasingly being used to evaluate hepatic functional reserve and operability in HAE patients with underlying liver disease [13–15].

Over the past few years, many attempts have also been made to formulate new criteria for the proper assessment of liver function, thereby ensuring safe liver resection [16, 17]. Recently, the Albumin-Indocyanine Green Evaluation (ALICE) grading system, which is calculated based on preoperative serum albumin level and ICG-R15 test results, was developed as an effective tool for evaluating the hepatic functional reserve and predicting the postoperative clinical outcomes in patients with hepatocellular carcinoma (HCC) [18]. And a high ALICE grade is associated with poor hepatic functional reserve, increased risk of postoperative complications, and high mortality [18, 19]. As a result, this grading system may also be a sensitive and precise preoperative assessment for surgical candidates of HAE.

As per the current literature, there are very few studies on the preoperative hepatic functional reserve in HAE patients, and a systematic comparative study of various predictive methods for their prognosis is needed. Therefore, the present study aimed to investigate the value of the ICG-R15 test and ALICE grading system in predicting severe posthepatectomy liver failure (PHLF) and postoperative mortality of patients with HAE undergoing radical hepatectomy.

## Patients and methods

### Patients

**Fig. 1:**
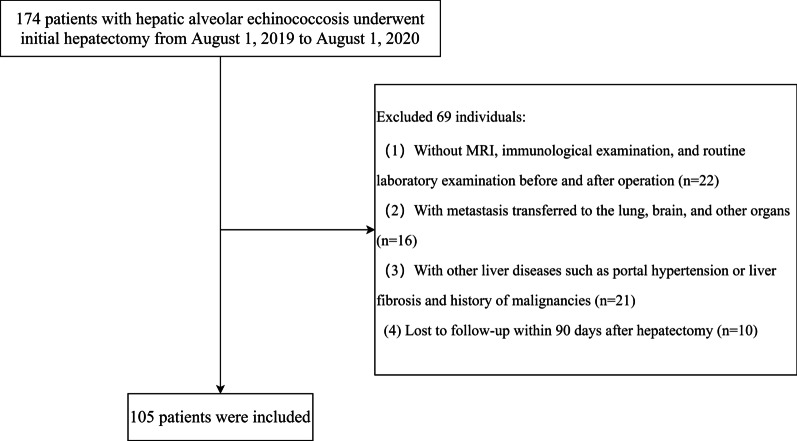
The flowchart of patients enrolled in this study

In this retrospective study, we reviewed HAE patients who underwent hepatectomy initially at the Sichuan Provincial People's Hospital between August 1, 2019, and August 1, 2020. A total of 174 patients were evaluated for the following inclusion criteria: 1) being diagnosed with metabolically active HAE after a magnetic resonance imaging (MRI), surgery, or pathological examination at this hospital; 2) never been operated upon for HAE; 3) having the WHO Informal Working Group on AE PNM stage of I/II; 4) having undergone a hepatectomy with complete resection of the AE lesion; 5) having no history of malignancies; 6) having true and complete case information. Patients were excluded if 1) metastasis had transferred to the lung, brain, or any other organ; 2) there were other liver diseases involved, such as portal hypertension or liver fibrosis; 3) there was no record of MRI, immunological examination, or routine laboratory examination before and after operation; 4) they were lost to follow-up within 90 days after the hepatectomy. Finally, a total of 105 patients were enrolled for the analyses (Fig. 1).

The experimental protocol was established, according to the ethical guidelines of the Helsinki Declaration and was approved by the Human Ethics Committee of Sichuan Academy of Medical Sciences & Sichuan Provincial People's Hospital (NO2021-456). Written informed consent was obtained from individual or guardian participants.

### Definitions and follow up

In this study, liver resection of three or more Couinaud segments was defined as major resection, and the resection of less than three Couinaud segments was called minor resection [20]. As suggested by the International Study Group of Liver Surgery (ISGLS), patients with an increased international normalized ratio (INR) and concomitant hyperbilirubinemia on or after the postoperative day 5 are considered as posthepatectomy liver failure (PHLF) [21]. PHLF is divided into three grades, viz. A, B, and C. Patients with PHLF grade A require no change in their clinical management, those with grade B require noninvasive treatments such as plasma or albumin transfusion, and those with grade C require invasive treatments such as intubation, mechanical ventilation, hemodialysis, or extracorporeal liver support [21]. For the purpose of our study, only grade B and grade C PHLF were categorized as severe PHLF [22].

All patients were followed up regularly after the operation. These postoperative follow-ups were performed at the first month after resection, every 3 to 4 months for the first two years, and then every six months subsequently, until death or dropout. Investigations done during these follow-ups included physical examination, serum biochemistry evaluation, and at least one abdominal scan, such as abdomen ultrasonography, abdominal computed tomography, MRI, or fluorodeoxyglucose-positron emission tomography (FDG-PET).

### Calculation of the Child–Pugh score and the ALICE grading system

The calculation of the Child–Pugh score combined liver biochemical indices with other clinical indices, such as serum albumin, serum bilirubin, prothrombin time, hepatic encephalopathy, and ascites [23]. The Child–Pugh classifications are divided into 3 grades, viz. grade A (5–6 points), grade B (7–9 points), and grade C (10–15 points).

The ALICE score was calculated using the equation for linear predictor, viz. linear predictor = (0.663 × log10 ICG R15 [%]) − (0.0718 × albumin [g/L]). And the ALICE grade was defined as ALICE grade 1 when the linear predictor value ≤  − 2.20, as ALICE grade 2 when the linear predictor value was between − 2.20 and − 1.39, and as ALICE grade 3 when the linear predictor value was >  − 1.39 [18].

### Preoperative measurement of the ICG-R15 test

All patients underwent an ICG retention test before the operation. After fasting for more than 4 h, the patient was placed in a supine position, and rapid injection of 0.5 mg/kg ICG was administered to the patient through a peripheral vein in the forearm. An optical probe connected to the patient’s nose was used to monitor plasma ICG concentrations, and the value of ICG-R15 was calculated using a Pulse Dye Density-Graph Analyzer (DDG-3300 K, Nihon Kohden, Tokyo, Japan).

### Statistical analysis

Categorical data were analyzed using either the χ2 test or the Fisher’s exact test. Continuous data were expressed as mean ± SD and compared between groups using the t-test or the Wilcoxon rank-sum test, as appropriate. Factors predicting severe PHLF were determined using univariate and multivariate logistic regression analysis. The predictive ability of the Child–Pugh grade, ICG-R15, and ALICE grading system for PHLF was determined from the area under the ROC (receiver operating characteristic) curve (AUC) based on the optimal cutoff. Statistical analyses were carried out using the software AKIBM SPSS Statistics 25.0 (IBM Corp., Armonk, NY, USA) and GraphPad Prism version 9.2.0 (GraphPad, CA, USA). A value of *P* < 0.05 was considered statistically significant.

## Results

### Baseline characteristics

The details of all the 105 patients enrolled in the study are summarized in Table 1. The mean age of patients undergoing hepatectomy for HAE was 39 ± 13 years, and 41.9% of them were male. Forty-five patients (42.9%) had hepatitis B virus infection, and their mean body mass index (BMI) was 22.2 ± 3.0. Child–Pugh grade A, B, C accounted for 76 (72.4%), 28 (26.7%), 1 (0.9%), respectively. According to the ALICE grading system, there were 57 patients (54.3%) in ALICE grade 1, 40 patients (38.1%) in ALICE grade 2, and 8 patients (7.6%) in ALICE grade 3. The mean value of the ICG-R15 was 6.1% ± 5.4%, and the mean operative time was 6.7 ± 3.1 h. Most patients (61.9%) had more than one AE lesion, while 40 (38.1%) of them had only one lesion. Surgical resections included 78 (74.3%) major resections and 27 (25.7%) minor resections.

**Table 1:** Baseline characteristics of the included 105 patients and comparison of factors between patients with and without severe PHLF

		Severe PHLF		
Characteristics	All patients (n = 105)	No (n = 71)	Yes (n = 34)	*P* value
Sex, male	44 (41.9%)	34 (47.9%)	10 (29.4%)	0.073
Age, year	39 ± 13	39 ± 13	41 ± 12	0.485
BMI, kg/m2	22.2 ± 3.0	22.5 ± 3.0	21.6 ± 3.0	0.156
Positive HBsAg	45 (42.9%)	30 (42.3%)	15 (44.1%)	0.857
Serum albumin, g/L	35.7 ± 4.5	36.9 ± 3.9	33.2 ± 4.7	< 0.001
Total bilirubin, μmol/L	15.4 ± 13.9	11.6 ± 9.0	23.2 ± 18.4	< 0.001
INR	1.10 ± 0.21	1.07 ± 0.22	1.15 ± 0.18	< 0.001
Platelet count, 10^9^/L	260 ± 91	262 ± 92	254 ± 90	0.706
Child–Pugh grade				< 0.001
A	76 (72.4%)	62 (81.6%)	14 (18.4%)	
B	28 (26.7%)	9 (32.1%)	19 (67.9%)	
C	1 (0.9%)	0 (0%)	1 (100%)	
ICG-R15, %	6.1 ± 5.4	4.1 ± 3.6	10.3 ± 6.3	< 0.001
ALICE grade				< 0.001
Grade 1	57 (54.3%)	53 (93.0%)	4 (7.0%)	
Grade 2	40 (38.1%)	17 (42.5%)	23 (57.5%)	
Grade 3	8 (7.6%)	1 (12.5%)	7 (87.5%)	
lesion size, cm	10.4 ± 5.0	8.9 ± 3.8	13.5 ± 5.9	< 0.001
operative time, hour	6.7 ± 3.1	5.6 ± 2.4	9.0 ± 3.0	< 0.001
Lesion number				0.003
Single	40 (38.1%)	34 (85.0%)	6 (15.0%)	
Multiple	65 (61.9%)	37 (56.9%)	28 (43.1%)	
Extent of hepatectomy				0.001
Minor resection	27 (25.7%)	25 (92.6%)	2 (7.4%)	
Major resection	78 (74.3%)	46 (59.0%)	32 (41.0%)	
Blood loss				< 0.001
< 500 mL	41 (39.0%)	37 (90.2%)	4 (9.8%)	
≥ 500 mL	64 (61.0%)	34 (53.1%)	30 (46.9%)	

*BMI* Body Mass Index; *INR* international normalized ratio; *ICG* indocyanine green; *ICG‐R15* ICG retention test at 15 min; *ACLICE score* Albumin-Indocyanine Green Evaluation score

### Outcomes

After hepatectomy, a total of 34 (32.4%) patients developed severe PHLF, including 4 (11.8%) patients with ALICE grade 1, 23 (67.6%) patients with ALICE grade 2, and 7 (20.6%) patients with ALICE grade 3, respectively. In addition, 88.2% of them were in the Child–Pugh grade B and the remaining 11.8% were in the Child–Pugh grade C. There was a considerable difference in the incidence of severe PHLF between the groups.

Of the 105 patients, a total of 5 (4.8%) patients died after 90 days of follow-up, including 4 (80.0%) patients with ALICE grade 2, and one (20.0%) patient with ALICE grade 3. The cause of death was severe PHLF in 5 cases. The difference between the two groups was not statistically significant (*P* = 0.834).

### Univariate and multivariate analyses to determine factors predicting severe PHLF

The patients with severe PHLF had higher levels of serum total bilirubin and INR, lower levels of serum albumin, higher Child–Pugh scores, higher ICG-R15 scores, higher ALICE grades, a larger extent of hepatectomy, higher amount of blood loss, bigger lesions, and higher operative time (Table 1). All differences were statistically significant (*P* < 0.05).

Univariate analysis showed that serum albumin, total bilirubin, Child–Pugh score, ICG-R15, lesion size, operation time, multiple lesions, major resection, and ALICE grades were all associated with severe PHLF. Multivariate analysis included significantly different factors between patients with and without severe PHLF and further revealed that the ALICE grade (odds ratio (OR), 16.076; 95% confidence interval (CI), 2.055–125.746; *P* = 0.008) and operative time (OR, 1.330; 95% CI, 1.027–1.723; *P* = 0.031) were independent factors for predicting severe PHLF (Table 2).

**Table 2:** Univariate and multivariate analyses to identify factors predicting severe PHLF

	Univariate analysis		Multivariate analysis	
Characteristics	Odds ratio (95% CI)	*P* value	Odds ratio (95% CI)	*P* value
Serum albumin, g/L	0.809 (0.722–0.907)	< 0.0001	1.280 (0.945–1.733)	0.111
Total bilirubin, μmol/L	1.075 (1.030–1.122)	0.001	1.050 (0.986–1.117)	0.130
INR	6.386 (0.698–58.393)	0.101		
Child–Pugh score	2.607 (1.634–4.159)	< 0.0001	0.985 (0.346–2.801)	0.977
ICG-R15, %	32.381 (8.454–124.026)	< 0.0001	1.095 (0.952–1.259)	0.206
Lesion size, cm	1.218 (1.103–1.346)	< 0.0001	0.969 (0.823–1.140)	0.704
operation time, hour	1.532 (1.278–1.837)	< 0.0001	1.331 (1.028–1.724)	0.030
Multiple lesions	4.288 (1.582–11.623)	0.004	2.032 (0.367–11.247)	0.417
Major resection	8.696 (1.922–39.335)	0.005	2.548 (0.275–23.582)	0.410
Blood loss ≥ 500 mL	8.162 (2.604–25.583)	< 0.0001	1.875 (0.362–9.706)	0.453
ALICE grade	13.936 (4.985–38.957)	< 0.0001	15.975 (2.054–124.234)	0.008

*INR* international normalized ratio; *ICG* indocyanine green; *ICG‐R15* ICG retention test at 15 min

### ROC analysis of the Child–Pugh grade, ICG-R15, and ALICE grade for severe PHLF

**Fig. 2:**
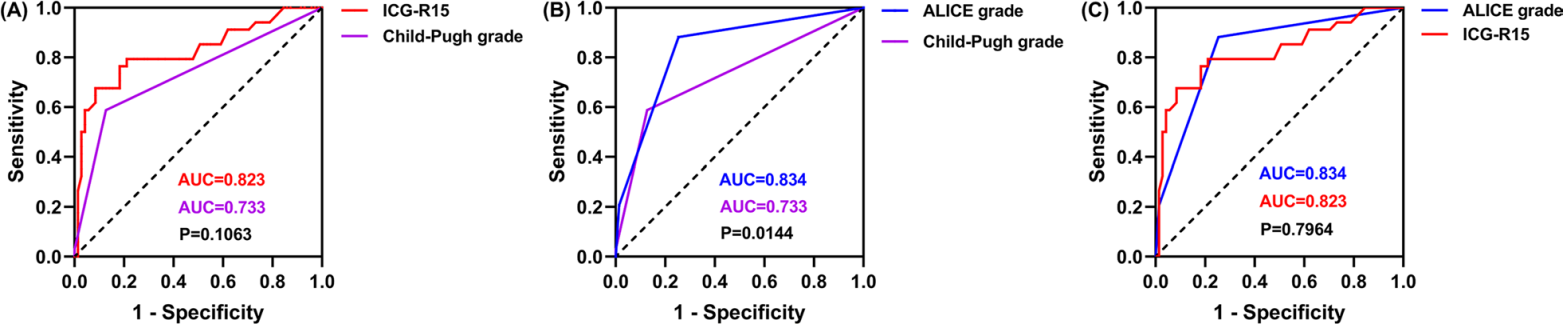
The ROC curves of the Child–Pugh grade, ICG-R15, and ALICE grade for predicting severe PHLF. **A** Child–Pugh grade (AUC, 0.733; 95% CI, 0.637–0.814) vs. ICG-R15 (AUC, 0.823; 95% CI, 0.73 vcv7–0.891). **B** ALICE grade (AUC, 0.834; 95% CI, 0.749–0.900) vs. Child–Pugh grade; **C** ALICE grade vs. ICG-R15. *ROC* receiver operating characteristic; *AUC* area under ROC curve; *CI* confidence interval; *ICG* indocyanine green; *ICG-R15* ICG retention test at 15 min; *ALICE grade* Albumin-Indocyanine Green Evaluation grade; *PHLF* posthepatectomy liver failure

The ROC and AUCs (95% CIs) of the indicators were analyzed to compare the predictive performance of ALICE grade along with Child–Pugh grade and ICG-R15 for predicting severe PHLF. Results showed that the ALICE grade had a greater predictive ability than Child–Pugh grade and ICG-R15. The AUC of ALICE grade for predicting severe PHLF was 0.834 (95% CI, 0.749–0.900). This value was significantly higher than that of the Child–Pugh grade (AUC, 0.733; 95% CI, 0.637–0.814; Fig. 2B, *P* = 0.0144). Further, the sensitivity and specificity of the ALICE grade in predicting severe PHLF were 88.2% and 74.6%, respectively. The AUC of ALICE grade for predicting severe PHLF was also higher than that of ICG-R15 (AUC, 0.823; 95% CI, 0.737–0.891; Fig. 2C), and the AUC of ICG-R15 for predicting severe PHLF was higher than that of Child–Pugh grade (Fig. 2A), although the differences were not statistically significant (*P* = 0.7964; *P* = 0.1063).

### Comparison of severe PHLF incidence and mortality between patients with different ICG-R15 and operative times

**Fig. 3:**
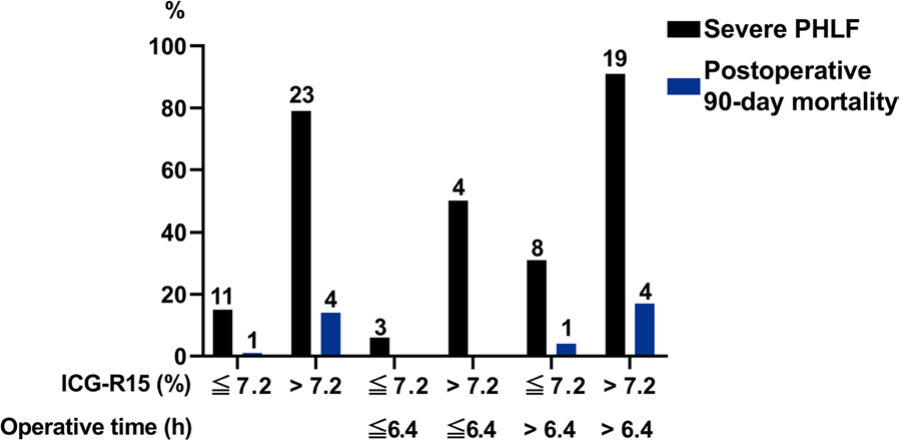
Incidence of severe PHLF and postoperative 90-day mortality between patients with different ICG-R15 and operative times. *ICG* indocyanine green; *ICG-R15* ICG retention test at 15 min; *PHLF* posthepatectomy liver failure. *Note* The number on the bar chart is the number of patients

With an optimal cutoff value of 7.2% and 6.4 h for PHLF, the sensitivity and specificity of ICG-R15 were 67.6% and 91.5%, and those for the operative time were 79.4% and 71.8%, respectively. All patients were divided into different groups based on the optimal cutoff value of ICG-R15 and operative time. The incidence of severe PHLF in patients with ICG-R15 > 7.2% was significantly higher than that in patients with ICG-R15 ≤ 7.2% (79.3% vs. 14.5%; *P* < 0.001; Fig. 3). Similar results were observed in the other two groups as well (operative time > 6.4 h: 90.5% vs. 30.8%; *P* < 0.001; operative time ≤ 6.4 h: 50.0% vs. 6.0%; *P* = 0.005; Fig. 3).

In addition, the incidence of mortality within the postoperative 90 days in patients with ICG-R15 > 7.2% was significantly higher than that of patients with ICG-R15 ≤ 7.2% (13.8% vs. 1.3%; *P* = 0.008; Fig. 3). For operative time greater than 6.4 h (> 6.4 h), the incidence of mortality in patients with ICG-R15 > 7.2% was also higher than that of patients with ICG-R15 ≤ 7.2%, although the difference was not statistically significant (19.0% vs. 3.8%; *P* = 0.108; Fig. 3). Moreover, when the operative time was under 6.4 h (≤ 6.4 h), there was no patient death after 12 months of follow-up.

### Comparison of severe PHLF incidence and mortality between patients with different ALICE grades and extents of hepatectomy

**Fig. 4:**
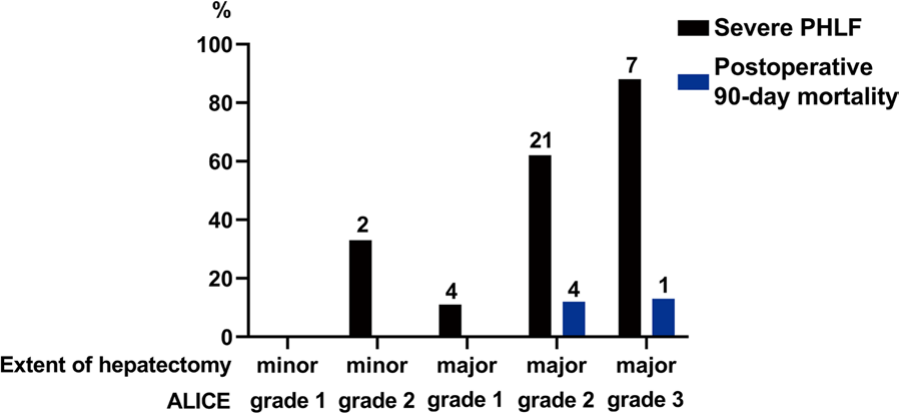
Incidence of severe PHLF and postoperative 90-day mortality between patients with different ALICE grades and extents of hepatectomy. *ALICE grade* Albumin-Indocyanine Green Evaluation grade; *PHLF* posthepatectomy liver failure. *Note* The number on the bar chart is the number of patients

As shown in Fig. 4, when minor resection was performed, the incidence of severe PHLF was significantly higher in patients with ALICE grade 2 than those with ALICE grade 1 (33.3% vs. 0.0%; *P* = 0.007). Similarly, in major resection, patients with higher ALICE grades had higher incidences of severe PHLF (ALICE grade 3 vs. grade 2 vs. grade 1; 87.5% vs. 61.8% vs. 11.1%).

In terms of the incidence of postoperative 90-day mortality, no patient died after minor resection during the follow-up. Interestingly, when major resection was performed, the incidence of postoperative 90-day mortality was about the same in both ALICE grades 2 and 3 (11.8% vs. 12.5%).

### Comparison of severe PHLF incidence and mortality between patients with different ALICE grades and Child–Pugh grades

**Fig. 5:**
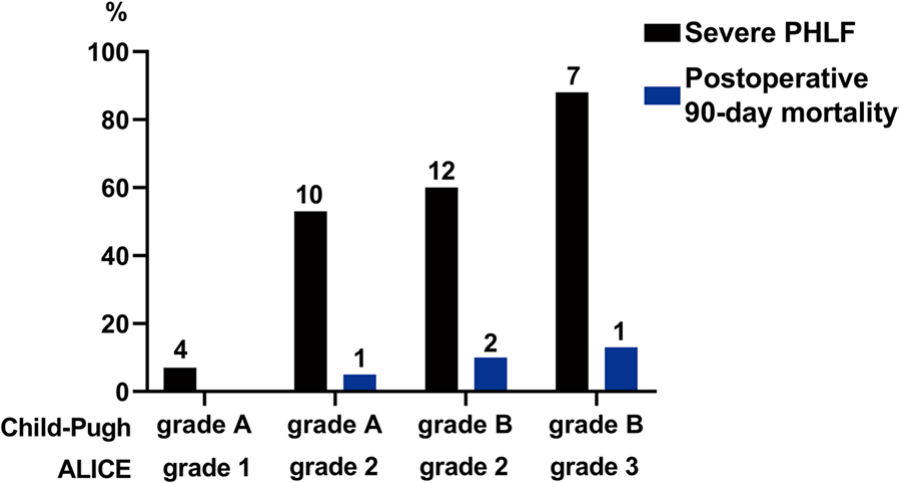
Incidence of severe PHLF and postoperative 90-day mortality between patients with different ALICE grades and Child–Pugh grades. *ALICE grade* Albumin-Indocyanine Green Evaluation grade; *PHLF* posthepatectomy liver failure. *Note* The number on the bar chart is the number of patients

The incidence of severe PHLF was significantly higher in patients with ALICE grade 2 than those with ALICE grade 1 within Child–Pugh grade A (52.6% vs. 7.0%; *P* < 0.001; Fig. 5). In Child–Pugh grade B, patients with ALICE grade 3 had a higher incidence of severe PHLF than those with ALICE grade 2 (87.5% vs. 60.0%; *P* = 0.167; Fig. 5). In Child–Pugh grade C, only one patient had severe PHLF, who was in ALICE grade 2.

The incidence of postoperative 90-day mortality was higher in patients with ALICE grade 2 than in those with ALICE grade 1 within Child–Pugh grade A, although the difference was not statistically significant (5.3% vs. 0.0%; *P* = 0.083; Fig. 5). However, within Child–Pugh grade B, the incidence of postoperative 90-day mortality was about the same in both ALICE grades 2 and 3 (12.5% vs. 10.0%; Fig. 5). In Child–Pugh grade C, the only patient who died was in ALICE grade 2.

## Discussion

In order to find the most suitable method for assessing the hepatic functional reserve in HAE patients before hepatectomy, this study was the first to compare the predictive value of Child–Pugh grade, ICG-R15, and ALICE grading system for clinical outcomes. As observed in this study, preoperative ICG-R15 and ALICE grading system were reliable predictors of severe PHLF and postoperative mortality in HAE patients. And patients with high ICG-R15 and ALICE grades had significantly higher severe PHLF incidence and postoperative mortality compared with those with low ICG-R15 and ALICE grades.

Many studies have shown that HAE is a disease characterized by infinite infiltrating growth and is therefore often considered to be a tumor-like disease [24]. In most cases, radical surgical resection of the entire parasitic mass is necessarily required [8, 25–27]. For multiple large lesions, radical hepatectomy, palliative hepatectomy, and liver transplantation are the three main surgical procedures [28]. Therefore, a precise evaluation of the liver function before the hepatectomy procedure can simply help surgeons choose the effective surgical treatment [14, 29].

The Child–Pugh grade, MELD score, and ICG-R15 are the three most widely used tools to assess liver function prior to hepatectomy [14, 15, 23, 29]. And in most Eastern countries, ICG-R15 was also the most common methods to select suitable HCC patients for liver resection [30, 31]. However, because some studies have recommended MELD score as the most suitable method for patients with severe cirrhosis, and the factors used to calculate MELD score, such as preoperative serum bilirubin, creatinine and INR, could also be easily affected by other non-hepatic factors, MELD score was not considered in the present study [32]. Moreover, it has been demonstrated that serum albumin is a strong predictor of the liver function and is also considered to be the most important variable influencing the Child–Pugh grade or the ALBI grade in HCC patients undergoing surgical resection [33, 34]. Considering the similarities between the two diseases, this result may also occur in HAE patients. Therefore, ALICE grading system, a combination of serum albumin and the ICG-R15, could be a simple and precise method for predicting postoperative outcomes in HAE hepatectomy.

As observed in univariate analysis, serum albumin, total bilirubin, Child–Pugh score, ICG-R15, lesion size, operation time, multiple lesions, major resection, and ALICE grade were all associated with severe PHLF. Multivariate analyses further validated that the ALICE grade was an independent factor in predicting severe PHLF, while the predictive values of Child–Pugh score and ICG-R15 were not statistically significant. These results preliminarily showed that preoperative ALICE grade had a better predictive value than Child–Pugh score and ICG-R15. In addition, ROC analysis revealed that the AUC of ALICE grade for predicting severe PHLF was higher than that of Child–Pugh grade and ICG-R15, which further demonstrated that the ALICE grade had a better predictive value than Child–Pugh grade and ICG-R15.

In this study, the optimal cutoff value of ICG-R15 for severe PHLF was 7.2%. The incidence of severe PHLF and postoperative 90-day mortality in patients with ICG-R15 > 7.2% were 79.3% and 13.8%, respectively, which were significantly higher than those of patients with ICG-R15 ≤ 7.2% (14.5% and 1.3%). However, when the operative time was less than 6.4 h (≤ 6.4 h), there was no patient died after 90 days of follow-up. Therefore, our results demonstrated that when the expected operation time is greater than 6.4 h, patients with ICG-R15 > 7.2% need to be carefully selected for hepatectomy.

In addition to liver functional reserve, the extent of liver resection is also one of the important factors for predicting postoperative liver function impairment and mortality. To better reflect the value of liver function assessment, we further combined the extent of liver resection with the ALICE grade in the analysis. In the present study, when minor resection was performed, the incidence of severe PHLF was relatively low, and no patient died within 90 days of follow-up. However, higher ALICE grades were associated with higher incidence of PHLF and postoperative mortality at the time of major resection. Therefore, we suggested that every HAE patient undergoing major resection should be graded first using the ALICE grading system to ensure a safe surgical option, and that major resection should be carefully selected for patients with high ALICE grades.

In Child–Pugh grade A, patients with ALICE grade 2 had a significantly higher incidence of severe PHLF and postoperative 90-day mortality than those with ALICE grade 1 (52.6% vs. 7.0%; 5.3% vs. 0%). And in Child–Pugh grade B, patients with ALICE grade 3 also had a higher incidence of severe PHLF than those with ALICE grade 2 (87.5% vs. 60.0%), while the postoperative 90-day mortality was similar (12.5% vs. 10.0%). Considering the high proportion of patients with Child–Pugh grade A (72.4%), ALICE grading system may allow further stratification of postoperative prognosis among HAE patients to guide the operation.

**Fig. 6:**
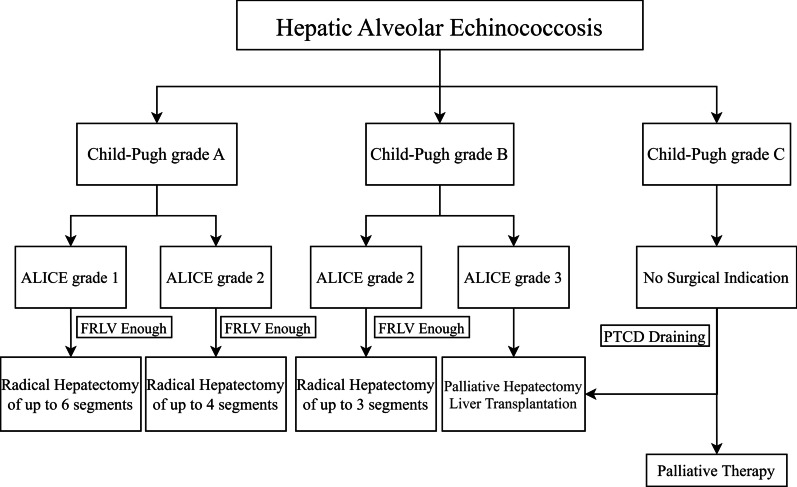
Flow diagrams of surgical guidelines for hepatic alveolar echinococcosis according to preoperative Child–Pugh grade and ALICE grading system. *PTCD* percutaneous transhepatic cholangial drainage; *ALICE grade* Albumin-Indocyanine Green Evaluation grade; *FRLV* future remnant liver volume

Since the 1990s, surgical decision-making algorithms based on ICG-R15 have been extensively studied and were first reported by Makuuchi et al. [33, 35]. Recently, a new surgical decision-making algorithm based on the ALICE grade was developed [18, 19]. This new system clearly stratified the postoperative risk in Child–Pugh A patients into three risk categories. In addition, many studies have also shown surgical approaches and simplified flow diagrams for definitive treatment of HAE [25, 26, 36]. Therefore, based on previous research results and the experience of our team, we developed a surgical decision-making flow diagram based on the Child–Pugh grade and ALICE grading system for HAE patients (Fig. 6). For patients with ALICE grade 1, since the risk of PHLF and mortality is extremely low, we recommended radical hepatectomy of up to 6 Couinaud’s segments when future remnant liver volume (FRLV) is sufficient. For patients with ALICE grade 2, when FRLV is enough, radical hepatectomy limited to 4 Couinaud’s segments is recommended, such as a right hepatectomy or an extended left hepatectomy. In Child–Pugh grade B, radical liver resection is recommended for up to 3 Couinaud’s segments for patients with ALICE grade 2. In addition, because most patients classified as ALICE grade 3 had severe PHLF, we recommended that they should be carefully selected for surgical resection. Palliative hepatectomy or liver transplantation could be suitable alternatives where the lesion status is appropriate. HAE patients with Child–Pugh grade C showed no indications for hepatectomy, but the application of percutaneous transhepatic cholangial drainage (PTCD) and palliative therapy could effectively reduce the liver damage, bacterial infection or life-threatening sepsis caused by biliary obstruction [37, 38].

However, there are some limitations in the present study. For example, only one patient with Child–Pugh grade C was selected for hepatectomy, and this may affect the reliability of the results. Moreover, this study included patients only from one center, and the sample size was relatively small. Therefore, further multicenter, large-scale prospective studies are needed to validate our findings.

## Conclusion

The ALICE grading system could provide a more precise and objective evaluation of the liver function for HAE patients. Although surgical decision-making in patients with HAE can be very challenging, it can be preliminarily determined using a combination of the preoperative Child–Pugh grade and ALICE grading system.

## Data Availability

The data that support the findings of this study are available from the corresponding author upon reasonable request.

## References

[1] Stojkovic M, Junghanss T. Chapter 26—Cystic and alveolar echinococcosis. In: Handbook of Clinical Neurology. Volume 114, edn. Edited by Garcia HH, Tanowitz HB, Del Brutto OH: Elsevier; 2013:327–334. 10.1016/B978-0-444-53490-3.00026-1 23829922

[2] Nunnari G, Pinzone MR, Gruttadauria S, Celesia BM, Madeddu G, Malaguarnera G, Pavone P, Cappellani A, Cacopardo B (2012). Hepatic echinococcosis: clinical and therapeutic aspects. World J Gastroenterol.

[3] Hatipoglu S, Bulbuloglu B, Piskin T, Kayaalp C, Yilmaz S (2013). Living donor liver transplantation for alveolar echinococcus is a difficult procedure. Transpl Proc.

[4] Joliat GR, Melloul E, Petermann D, Demartines N, Gillet M, Uldry E, Halkic N (2015). Outcomes after liver resection for hepatic alveolar echinococcosis: a single-center cohort study. World J Surg.

[5] Wang X, Dai G, Li M, Jia W, Guo Z, Lu J (2020). Prevalence of human alveolar echinococcosis in China: a systematic review and meta-analysis. BMC Public Health.

[6] Wen H, Vuitton L, Tuxun T, Li J, Vuitton DA, Zhang W, McManus DP. Echinococcosis: advances in the 21st Century. Clin Microbiol Rev 2019;32(2). 10.1128/CMR.00075-18 PMC6431127 30760475

[7] Pohnan R, Ryska M, Hytych V, Matej R, Hrabal P, Pudil J (2017). Echinococcosis mimicking liver malignancy: a case report. Int J Surg Case Rep.

[8] Yang M, Su W, Deng X, Deng J, Li P, Li X (2017). Enhanced recovery after surgery program in patients from Tibet Plateau undergoing surgeries for hepatic alveolar echinococcosis. J Surg Res.

[9] Hong AJ, Zheng SS, Ping CX, Feng DK, Jia F, Ping B, Ping GX, Ping LW. Consensus on evaluation of hepatic functional reserve before hepatectomy (2011 edition). 2015.

[10] Vauthey JN, Klimstra D, Franceschi D, Tao Y, Brennan M: Vauthey JN, Klimstra D, Franceschi D, Tao Y, Fortner J, Blumgart L, Brennan M. Factors affecting long-term outcome after hepatic resection for hepatocellular carcinoma. Am J Surg 169: 28–35. The American Journal of Surgery 1995, 169(1):28–34; discussion 34–25. 10.1016/s0002-9610(99)80106-8 7817995

[11] Teh SH, Christein J, Donohue J, Que F, Kendrick M, Farnell M, Cha S, Kamath P, Kim R, Nagorney DM (2005). Hepatic resection of hepatocellular carcinoma in patients with cirrhosis: model of end-stage liver disease (MELD) score predicts perioperative mortality. J Gastrointest Surg.

[12] Delis SG, Bakoyiannis A, Biliatis I, Athanassiou K, Tassopoulos N, Dervenis C. Model for end‐stage liver disease (MELD) score, as a prognostic factor for post‐operative morbidity and mortality in cirrhotic patients, undergoing hepatectomy for hepatocellular carcinoma. HPB 2009;11(4). 10.1111/j.1477-2574.2009.00067.x PMC2727090 19718364

[13] Iimuro Y (2017). ICG clearance test and 99mTc-GSA SPECT/CT fusion images. Vis Med.

[14] Forner A, Llovet JM, Bruix J (2012). Hepatocellular carcinoma. Lancet (London, England).

[15] Schneider PD (2004). Preoperative assessment of liver function. Surg Clin N Am.

[16] Donadon M, Costa G, Cimino M, Procopio F, Fabbro DD, Palmisano A, Torzilli G (2015). Safe hepatectomy selection criteria for hepatocellular carcinoma patients: a validation of 336 consecutive hepatectomies. The BILCHE score. World J Surg.

[17] Ichikawa T, Uenishi T, Takemura S, Oba K, Ogawa M, Kodai S, Shinkawa H, Tanaka H, Yamamoto T, Tanaka S (2009). A simple, noninvasively determined index predicting hepatic failure following liver resection for hepatocellular carcinoma. J Hepatobiliary Pancreat Surg.

[18] Takashi K, Kiyoshi H, Katsumi A, Emilie U, Chikara S, Takamune Y, Junichi A, Junichi K, Nobuhisa A, Yoshihiro S (2016). Assessment of preoperative liver function in patients with hepatocellular carcinoma—The Albumin-Indocyanine Green Evaluation (ALICE) Grade. PLoS ONE.

[19] Kokudo T, Hasegawa K, Shirata C, Tanimoto M, Halkic N (2019). Assessment of preoperative liver function for surgical decision making in patients with hepatocellular carcinoma. Liver Cancer.

[20] Pol B, Campan P, Hardwigsen J, Botti G, Pons J, Le Treut YP (1999). Morbidity of major hepatic resections: a 100-case prospective study. Eur J Surg Acta chirurgica.

[21] Rahbari NN, Garden OJ, Padbury R, Brooke-Smith M, Crawford M, Adam R, Koch M, Makuuchi M, Dematteo RP, Christophi C (2011). Posthepatectomy liver failure: a definition and grading by the International Study Group of Liver Surgery (ISGLS). Surgery.

[22] Wang YY, Zhao XH, Ma L, Ye JZ, Wu FX, Tang J, You XM, Xiang BD, Li LQ (2018). Comparison of the ability of Child-Pugh score, MELD score, and ICG-R15 to assess preoperative hepatic functional reserve in patients with hepatocellular carcinoma. J Surg Oncol.

[23] Durand F, Valla D. Assessment of the prognosis of cirrhosis: Child–Pugh versus MELD. J Hepatol 2005;42(1-supp-S):S100–S107. 10.1016/j.jhep.2004.11.015 15777564

[24] Kern P, Menezes da Silva A, Akhan O, Müllhaupt B, Vizcaychipi KA, Budke C, Vuitton DA. The Echinococcoses: diagnosis, clinical management and burden of disease. Adv Parasitol 2017;96:259–369. 10.1016/bs.apar.2016.09.006 28212790

[25] Yang C, He J, Yang X, Wang W (2019). Surgical approaches for definitive treatment of hepatic alveolar echinococcosis: results of a survey in 178 patients. Parasitology.

[26] Chen KF, Tang YY, Wang R, Fang D, Chen JH, Zeng Y, Li B, Wen TF, Wang WT, Wu H (2018). The choose of different surgical therapies of hepatic alveolar echinococcosis: A single-center retrospective case-control study. Medicine.

[27] Hillenbrand A, Gruener B, Kratzer W, Kern P, Graeter T, Barth TF, Buttenschoen K, Henne-Bruns D (2017). Impact of safe distance on long-term outcome after surgical therapy of alveolar echinococcosis. World J Surg.

[28] Vuitton DA, Azizi A, Richou C, Vuitton L, Blagosklonov O, Delabrousse E, Mantion GA, Bresson-Hadni S (2016). Current interventional strategy for the treatment of hepatic alveolar echinococcosis. Expert Rev Anti Infect Ther.

[29] de Lope CR, Tremosini S, Forner A, Reig M, Bruix J (2012). Management of HCC. J Hepatol.

[30] Lisotti A, Azzaroli F, Buonfiglioli F, Montagnani M, Cecinato P, Turco L, Calvanese C, Simoni P, Guardigli M, Arena R (2014). Indocyanine green retention test as a noninvasive marker of portal hypertension and esophageal varices in compensated liver cirrhosis. Hepatology (Baltimore, MD).

[31] Fonseca AL, Cha CH (2014). Hepatocellular carcinoma: a comprehensive overview of surgical therapy. J Surg Oncol.

[32] Kim WR, Biggins SW, Kremers WK, Wiesner RH, Kamath PS, Benson JT, Edwards E, Therneau TM (2008). Hyponatremia and mortality among patients on the liver-transplant waiting list. N Engl J Med.

[33] Seyama Y, Kokudo N (2009). Assessment of liver function for safe hepatic resection. Hepatol Res.

[34] Johnson PJ, Berhane S, Kagebayashi C, Satomura S, Teng M, Reeves HL, O'Beirne J, Fox R, Skowronska A, Palmer D (2015). Assessment of liver function in patients with hepatocellular carcinoma: a new evidence-based approach-the ALBI grade. J Clin Oncol.

[35] Makuuchi M, Kosuge T, Takayama T, Yamazaki S, Kakazu T, Miyagawa S, Kawasaki S (1993). Surgery for small liver cancers. Semin Surg Oncol.

[36] Du C, Liu Z, Yang X, Yan L, Li B, Wen T, Yang J, Xu M, Chen Z, Wang W (2016). Hepatectomy for patients with alveolar echinococcosis: Long-term follow-up observations of 144 cases. Int J Surg (London, England).

[37] Takada T (2001). Is preoperative biliary drainage necessary according to evidence-based medicine?. J Hepatobiliary Pancreat Surg.

[38] Sato N, Namieno T, Takahashi H, Yamashita K, Matsuhisa T, Aoki S, Uchino J (1996). A long-surviving patient with recurrences of hepatic alveolar echinococcosis after traumatic intra-abdominal rupture. J Gastroenterol.

